# Supplementary material for: Admission Point-of-Care Testing for the Clinical Care of Children with Cerebral Malaria
Source: Trop Med Infect Dis. 2024 Sep 11;9(9):210. doi: 10.3390/tropicalmed9090210 (PMC11435513; doi:10.3390/tropicalmed9090210)
Supplement: Supplementary file 1 [file tropicalmed-09-00210-s001.zip › tropicalmed-3190635-supplementary.pdf]

Table S1. Normal ranges for Point of Care Testing and Laboratory-Based Testing.

Value	Normal Reference Range
Point of care testing results	
Glucose	3.0 - 6.0 mmol/L
Lactate	1.0 - 3.3 mmol/L
Creatinine	
2 months - 3 years	0.2 - 0.5 mg/dL
3 years - 10 years	0.3 - 0.7 mg/dL
≥10 years	0.3 - 0.9 mg/dL
pH	7.35 - 7.45
pCO2 - PoCT	35 - 45 mmHg
Base Excess	-2 to +3
Bicarbonate - PoCT	22 - 26 mmol/L
Sodium - PoCT	136 - 145 mmol/L
Potassium - PoCT	3.3 - 4.6 mmol/L
iCa	1.12 - 1.32 mmol/L
Laboratory-based testing results	
pCO2	35 - 45 mmHg
Bicarbonate	21 - 28 mmol/L
Sodium	126 - 145 mmol/L
Potassium	3.5 - 4.3 mmol/L
Total Ca	2.00 - 2.40 mmol/L

Table S2. Comparison of point of care testing results with laboratory-based testing results.

Test result	Point of care testing results	Laboratory-based testing results	p-value for difference
Bicarbonate (mmol/L)	19.00(16.00,22.00)	16.00(12.00,18.00)	1.154e-10
Sodium (mmol/L)	141.00 (137.80,144.00)	138.00(135.00,141.00)	2.145e-5
Potassium (mmol/L)	4.600(4.100,4.900)	4.500(4.000,5.3250)	7.076e-1
Calcium (mmol/L) – total vs. ionized	61 (60.39%) (proportion in the normal range)	118 (69.82%) (proportion in the normal range)	1.464e-1
